# Urinary Plasmids Reduce Permissivity to Coliphage Infection

**DOI:** 10.1128/spectrum.01309-23

**Published:** 2023-07-06

**Authors:** Cesar Montelongo Hernandez, Catherine Putonti, Alan J. Wolfe

**Affiliations:** a Department of Microbiology and Immunology, Stritch School of Medicine, Loyola University Chicago, Maywood, Illinois, USA; b Bioinformatics Program, Loyola University Chicago, Chicago, Illinois, USA; c Department of Biology, Loyola University Chicago, Chicago, Illinois, USA; LSU Health New Orleans

**Keywords:** bacteriophages, plasmids, urinary tract, microbiota, drug resistance, microbial, human microbiome, multidrug resistance, urinary tract infection

## Abstract

The microbial community of the urinary tract (urinary microbiota or urobiota) has been associated with human health. Bacteriophages (phages) and plasmids present in the urinary tract, like in other niches, may shape urinary bacterial dynamics. While urinary Escherichia coli strains associated with urinary tract infection (UTI) and their phages have been catalogued for the urobiome, bacterium-plasmid-phage interactions have yet to be explored. In this study, we characterized urinary E. coli plasmids and their ability to decrease permissivity to E. coli phage (coliphage) infection. Putative F plasmids were predicted in 47 of 67 urinary E. coli isolates, and most of these plasmids carried genes that encode toxin-antitoxin (TA) modules, antibiotic resistance, and/or virulence. Urinary E. coli plasmids, from urinary microbiota strains UMB0928 and UMB1284, were conjugated into E. coli K-12 strains. These transconjugants included genes for antibiotic resistance and virulence, and they decreased permissivity to coliphage infection by the laboratory phage P1vir and the urinary phages Greed and Lust. Plasmids in one transconjugant were maintained in E. coli K-12 for up to 10 days in the absence of antibiotic resistance selection; this included the maintenance of the antibiotic resistance phenotype and decreased permissivity to phage. Finally, we discuss how F plasmids present in urinary E. coli strains could play a role in coliphage dynamics and the maintenance of antibiotic resistance in urinary E. coli.

**IMPORTANCE** The urinary tract contains a resident microbial community called the urinary microbiota or urobiota. Evidence exists that it is associated with human health. Bacteriophages (phages) and plasmids present in the urinary tract, like in other niches, may shape urinary bacterial dynamics. Bacterium-plasmid-phage interactions have been studied primarily in laboratory settings and are yet to be thoroughly tested in complex communities. This is especially true of the urinary tract, where the bacterial genetic determinants of phage infection are not well understood. In this study, we characterized urinary E. coli plasmids and their ability to decrease permissivity to E. coli phage (coliphage) infection. Urinary E. coli plasmids, encoding antibiotic resistance and transferred by conjugation into naive laboratory E. coli K-12 strains, decreased permissivity to coliphage infection. We propose a model by which urinary plasmids present in urinary E. coli strains could help to decrease phage infection susceptibility and maintain the antibiotic resistance of urinary E. coli. This has consequences for phage therapy, which could inadvertently select for plasmids that encode antibiotic resistance.

## INTRODUCTION

The urinary tract of asymptomatic, presumably healthy, individuals is not sterile. It contains microbiota, including bacteria, eukaryotic viruses, fungi, archaea, and bacteriophages (1, 2). The presence and proportion of specific bacteria in the urinary microbiota (urobiota) are linked to both asymptomatic and symptomatic urinary conditions (1, 3). Phages are key influencers of bacterial communities and, by extension, human health (4–6). Urinary phages are rich and diverse, both free-living and as prophages (7, 8). As in other anatomical sites, urinary phages likely impact urobiota populations by influencing population structure, genetic exchange, and bacterial metabolism (4, 9–11). In contrast, a bacterium may modulate a phage’s life cycle via traits encoded by its chromosome or by mobile genetic elements (MGEs) such as plasmids (12–15).

Plasmids can influence phage-bacterium dynamics by transmitting traits vertically and horizontally in bacterial populations (16–18). For example, TraT, a component of the *tra* operon in Escherichia coli, can block both foreign plasmids and phage invasion (16, 19, 20). Toxin-antitoxin (TA) systems make plasmid loss and, by extension, the antitoxin lethal to the host, yet TA modules can also antagonize phage life cycles (17, 21, 22). In contrast, phages may target plasmid components such as the conjugal apparatus plasmids used to transfer copies of themselves (23–25). This can cause plasmid loss and result in antibiotic sensitivity as many plasmids carry antibiotic resistance determinants (23, 25–27). Bacterium-plasmid-phage interactions have been studied primarily in laboratory settings and are yet to be thoroughly tested in complex communities. Bacterium-plasmid-phage interactions are multifaceted and could be key to understanding the dynamics and evolution of environmental microbial populations such as the urobiota (13, 28, 29). A key unanswered question is whether urinary plasmids and phages interact.

The best-studied bacterium of the urinary tract is E. coli, often associated with urinary tract infection (UTI) (30, 31). Malki et al. isolated seven E. coli phages (coliphages) from urine collected via catheterization from women with urge urinary incontinence (32). Two of these coliphages, Greed and Lust, infect and lyse E. coli strains (8). Phage predation of E. coli in the urinary tract remains understudied, including the role that plasmids play in urinary bacterium-phage dynamics (8, 33–36). Previously, we described plasmids present in urinary E. coli isolates; most were F plasmids with genes for conjugation (37, 38). F plasmids are genetically heterogeneous and easily transmissible plasmids with high clinical relevance as they often carry genes encoding antibiotic resistance and virulence (39–41).

We hypothesized that urinary E. coli F plasmids could influence permissivity to phage infection; we tested this hypothesis by monitoring lysis by coliphages P1vir, Greed, and Lust. P1vir is a laboratory phage mutated to enter only the lytic cycle, and Greed and Lust are urinary phages that lyse E. coli, including some urinary strains (8, 32, 33, 42). We found F plasmids in most of our urinary E. coli isolates; they often carried genes associated with TA modules, antibiotic resistance, and/or virulence. We used conjugation to transfer plasmids from their native hosts to laboratory E. coli K-12 strains; the resultant transconjugants had reduced permissivity to infection by P1vir, Greed, and Lust and now contained antibiotic resistance and virulence genes. One transconjugant was stable, retaining its antibiotic resistance for 10 days without plasmid selection. We thus propose that urinary plasmids protect urinary E. coli from phage predation, maintaining antibiotic resistance in the population (43–45).

## RESULTS

### Urinary E. coli plasmid sequence analysis.

Of the 67 urinary E. coli genomic raw sequence reads, 57 contained putative plasmid sequences (see Table S1 in the supplemental material). We sought urinary E. coli isolates likely to carry F plasmids (even if those isolates carried other plasmids); therefore, for this initial component of the project, plasmidic assemblies in each isolate were treated as a singular plasmidic unit and analyzed for their F plasmid content. All manually curated plasmidic assemblies had homology to plasmid entries in the NCBI database (a query coverage of 71 to 100% with a sequence identity of 96 to 100%) (Table S1). The homology of plasmidic assemblies was primarily to E. coli plasmid entries but also to plasmids from other members of the family *Enterobacteriaceae*, including Klebsiella, *Shigella*, and Enterobacter. Two plasmid assemblies shared homology with the plasmid in uropathogenic E. coli (UPEC) strain UTI89.

Plasmidic assemblies were scanned for plasmid incompatibility genes (Table S2). Fifty-seven urinary E. coli isolates were predicted to carry *inc* genes organized into two groups: those containing either at least one *incF* gene (IncF group) (*n* = 47) or no *incF* genes (non-IncF group) (*n* = 10) (Table S2). While non-*incF* genes were identified in plasmidic assemblies, the most common incompatibility genes were from *incF*, which are associated with F plasmids (present in 68.7% of all profiled urinary E. coli strains and 82.5% of those strains predicted to carry plasmids). We compared the overall sequence similarities of the urinary plasmidic assemblies; the IncF group clustered into multiple subgroups. The plasmidic assemblies of the IncF group were predicted to possess a total of 2,060 unique open reading frames (ORFs), while the non-IncF group had a total of 895 unique ORFs. Only ~24% of all plasmid ORFs were assigned a known function. The IncF group had the highest count of distinct ORFs with assigned functions, including annotations for plasmid replication machinery, metal transport and resistance genes, leukotoxin genes, multidrug transporters, phage genes, and virulence regulators (data not shown).

**FIG 1:**
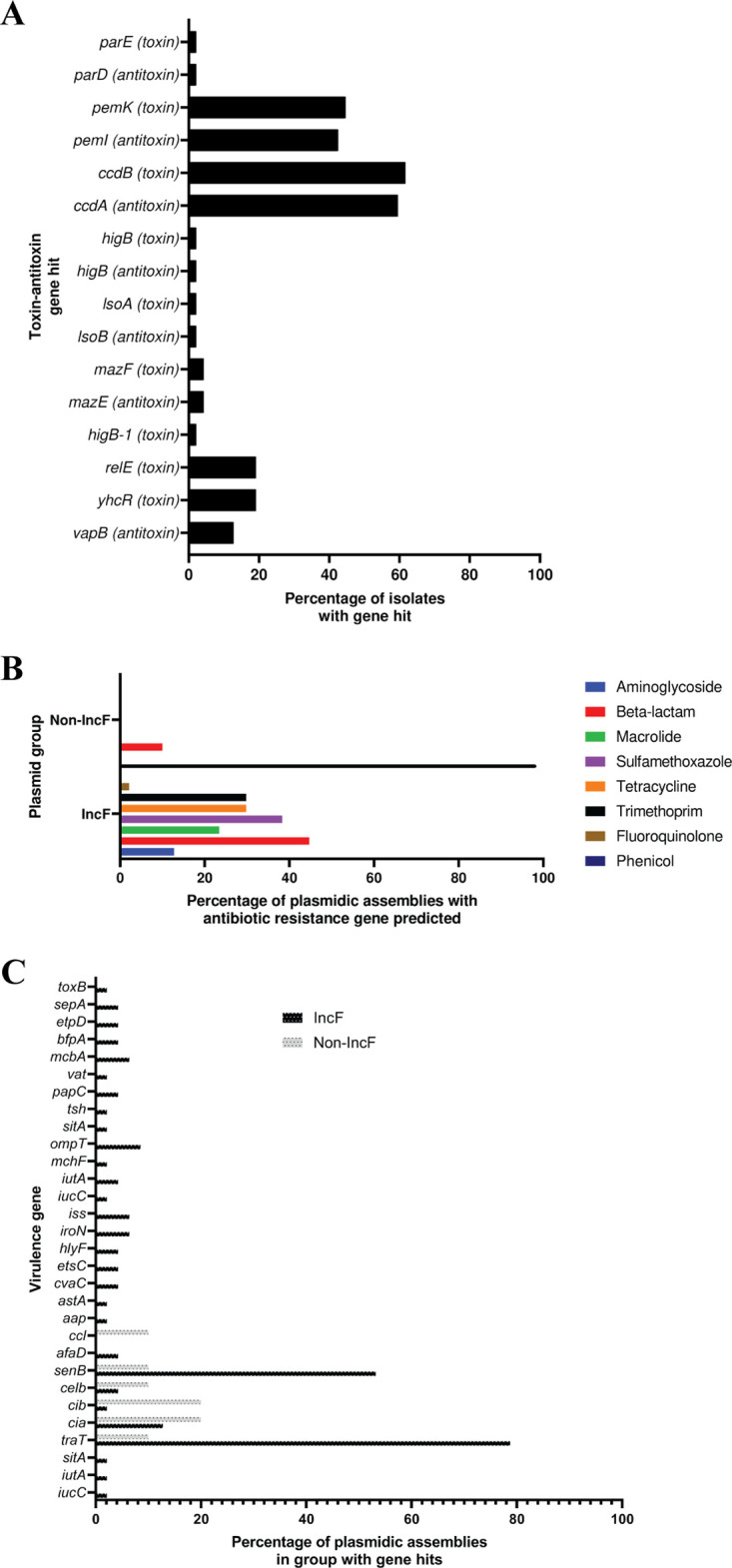
Proportions of plasmid addiction, antibiotic resistance, and virulence genes in urinary E. coli plasmidic assemblies (IncF = 47 plasmidic assemblies; non-IncF = 11 plasmidic assemblies). Percentages denote the plasmidic assembly Inc groups with the gene over that Inc group’s plasmidic assembly total. (A) Urinary E. coli F plasmid assemblies have a variety of TA genes that are associated with plasmid retention. *ccdAB* and *pemIK* are the most common modules. TA genes were not found in non-IncF plasmidic assemblies. (B) Types of antibiotic resistance predicted in plasmid types in urinary E. coli strains. IncF group plasmidic assemblies have a higher proportion of antibiotic resistance genes than non-IncF group plasmidic assemblies. (C) Percentage of isolates from each plasmid group predicted to have a given virulence gene. IncF group plasmidic assemblies had the largest variety and proportion of virulence gene hits. The most common virulence genes were *traT* (blocks invading plasmids) and *senB* (F plasmid-linked enterotoxin).

Plasmidic assemblies were profiled for TA, antibiotic resistance, and virulence genes. Sixteen TA genes were predicted via Prokka annotation in the IncF group plasmidic assemblies, but none were predicted in the non-IncF group. Complete TA pairs were identified for *ccdAB*, *isoAB*, *mazEF*, *parDE*, and *pemIK*; *ccdAB* and *pemIK* were the most frequent, with some plasmidic assemblies having hits for both modules (Fig. 1A). The plasmidic assemblies were predicted to confer resistance to the following antibiotics: aminoglycosides, fluoroquinolones, macrolides, streptomycin, sulfonamides, tetracycline (Tet), and trimethoprim (Fig. 1B). Some plasmidic assemblies were predicted to have no antibiotic resistance genes; in contrast, four F plasmidic assemblies (from urinary microbiota strains UMB0906, UMB0949, UMB3538, and UMB5924) were predicted to have seven antibiotic resistance genes (Fig. S1A). Many of the strains were predicted to grow on one or more antibiotics due to their plasmid(s) (Fig. S1B and C). Thirty distinct virulence genes were predicted in the plasmidic assemblies (Fig. 1C), with *traT* (78.72%) and *senB* (53.19%) being the most common in the IncF group. Non-IncF plasmidic assemblies had hits primarily to colicin-related virulence genes (*ccl*, *celb*, *cib*, and *cia*), which are signature genes of colicin plasmids, although the non-IncF plasmidic assembly from UMB0731 had hits to *traT* and *senB*.

### Urinary plasmid transconjugant phenotype testing.

To obtain a visual reference for future assays, we spotted lawns of E. coli K-12 derivatives with the lytic phages P1vir, Greed, and Lust; for controls, we used the temperate phage Lambda and lysogeny broth (LB). P1vir, Greed, and Lust resulted in cleared zones, while Lambda caused a turbid phenotype, and LB had no effect. We conjugated putative F plasmids from urinary E. coli strains (UMB0928, UMB1091, UMB1223, UMB1284, and UMB6721) to multiple MG1655-derived strains (MG1655/pCA24n-cm and MG1655 Δ*cobB yfiQ*::Cm), as described in Materials and Methods (38). This conjugation generated E. coli K-12 transconjugants containing plasmidic assemblies pU0928, pU1091, pU1223, pU1284, and pU6721. We then tested the susceptibilities of urinary E. coli strains and the transconjugants. The urinary plasmid donor strains were not permissive to the phages at any of the titers tested (Table 1). In contrast, the naive recipients were susceptible at every titer tested, including dilution by 8 orders of magnitude to a multiplicity of infection (MOI) of ~10^−6^ (10^2^ PFU/mL; 10^8^ CFU/mL). When exposed to phage, the phenotypes of the transconjugants carrying plasmidic assembly pU1091, pU1223, or pU6721 resembled those of their naive parent. In contrast, transconjugants carrying plasmidic assembly pU0928 or pU1284 were susceptible only at the highest titers of P1vir, Greed, and Lust, but there was no observable clearing after dilution of the phage concentration by 3 to 4 orders (i.e., decreased permissivity to infection). Multiple MG1655 derivatives were tested as recipients, with the results being consistent for all recipients tested. Given that pU0928 and pU1284 changed the spot titration phenotype, we further tested these plasmids, using pU1223 as the negative control.

**TABLE 1:** Phage spot titration results for E. coli^*a*^

Origin or genetic background	Strain designation	Mutation(s)	ASKA plasmid	Bladder plasmid	Marker(s)	Titration		
						P1vir	Lust	Greed
Urinary	UMB0928	NA	None	pU0928	Tc	No spot	No spot	No spot
Urinary	UMB1091	NA	None	pU1091	Tc	No spot	No spot	No spot
Urinary	UMB1223	NA	None	pU1223	Tc	No spot	No spot	No spot
Urinary	UMB1284	NA	None	pU1284	Tc	No spot	No spot	No spot
Urinary	UMB6721	NA	None	pU6721	Tc	No spot	No spot	No spot
MG1655	AJW1776	WT	None	None	None	8	8	8
MG1655	AJW4793	WT	pCA24n-Empty	None	Cm	8	8	8
MG1655	AJW5116	*yfiQ*::Cm, *cobB*::FRT	None	None	Cm	8	8	8
MG1655	AJW4793	WT	pCA24n-Empty	pU0928	Cm, Tc	3	4	3
MG1655	AJW4793	WT	pCA24n-Empty	pU1091	Cm, Tc	8	8	8
MG1655	AJW4793	WT	pCA24n-Empty	pU1223	Cm, Tc	8	8	8
MG1655	AJW4793	WT	pCA24n-Empty	pU1284	Cm, Tc	3	4	3
MG1655	AJW4793	WT	pCA24n-Empty	pU6721	Cm, Tc	8	8	8
MG1655	AJW5035	*yfiQ*::Kn	None	None	Kn	8	8	8
MG1655	AJW1776	WT	pCA24n-*yfiQ*	None	Cm	8	8	8
MG1655	AJW5035	*yfiQ*::Kn	pCA24n-*yfiQ*	None	Kn, Cm	7	8	8
MG1655	AJW5035	*yfiQ*::Kn	pCA24n-Empty	None	Kn, Cm	7	8	8
MG1655	AJW5184	*yfiQ*::Cm	None	None	Cm	8	8	8
MG1655	AJW5035	*yfiQ*::Kn	pCA24n-*yfiQ*	pU0928	Cm, Kn, Tc	3	4	3
MG1655	AJW5035	*yfiQ*::Kn	pCA24n-Empty	pU0928	Cm, Kn, Tc	4	5	4
MG1655	AJW5184	*yfiQ*::Cm	None	pU0928	Cm, Tc	3	3	3
BW25113	AJW4688	*yfiQ*::Kn	None	None	Kn, Tc	8	8	8
BW25113	AJW4688	*yfiQ*::Kn	None	pU0928	Kn, Tc	3	3	3
MG1655	AJW5037	*cobB*::Cm	None	None	Cm	8	8	8
MG1655	AJW5037	*cobB*::Cm	None	pU0928	Cm, Tc	2	3	3

a Note that *yfiQ* and *cobB* mutations do not affect the phenotype under the growth conditions utilized. Numbers in the phage columns denote the titration of phage (starting at 10^9^ PFU/mL, which is titration 1) at which lysis was observed on an E. coli lawn (~10^8^ CFU/mL). A higher number indicates lysis observed at a higher titration (i.e., lower phage PFU per milliliter). NA, not applicable; WT, wild type; FRT, FLP recombination target.

**FIG 2:**
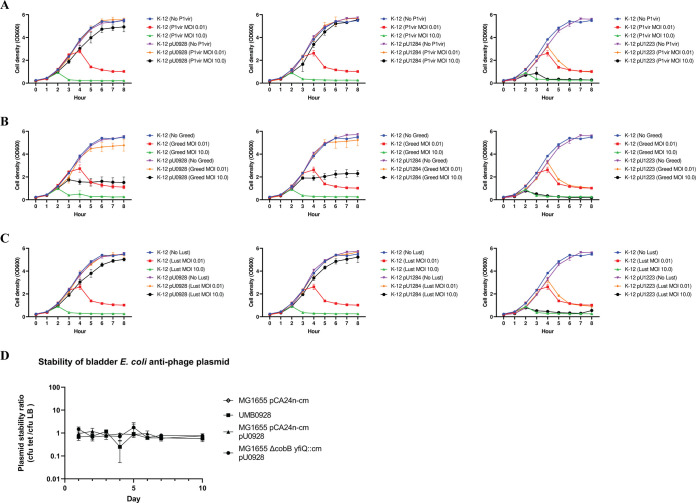
Growth of E. coli K-12 infected with phage and stability of urinary plasmids. (A to C) E. coli K-12 transconjugants (MG1655/pCA24n-cm) with pU0928 (left), pU1284 (middle), or pU1223 (right) infected with phage P1vir (A), Greed (B), and Lust (C) (MOI of 0.01 or 10.0). The growth of naive K-12 decreases at MOIs of 0.01 and 10.0. pU0928 and pU1284 decreased permissivity to phage infection, but pU1223 did not. Similar results were observed when a different K-12 strain (MG1655 Δ*cobB yfiQ*::Cm) was used (see Fig. S2A to C in the supplemental material). (D) Urinary isolate UMB0928 and E. coli K-12 variants were grown in the absence of antibiotic selection for plasmid pU0928 for 10 days. Cultures were plated onto tetracycline (pU0928 selection marker) and LB plates daily. A plasmid stability ratio (CFU on tetracycline plates divided by CFU on LB plates) of 1 indicates plasmid retention, while a ratio close to 0 indicates the loss of the plasmid. The negative control MG1655/pCA24n-cm without pU0928 did not grow on tetracycline plates.

Growth curves were used to assess the effect of the urinary plasmids on E. coli growth during phage infection at MOIs of 0, 0.01, and 10.0. P1vir infection of transconjugants with pU0928 or pU1284 but not pU1223 resulted in optical densities comparable to those of the controls uninfected with phage at all time points (Fig. 2A and Fig. S2A). Greed infection of pU0928 and pU1284 but not pU1223 transconjugants at an MOI of 0.01 resulted in growth like that of the uninfected control (Fig. 2B and Fig. S2B). Increasing the MOI of Greed to 10.0 resulted in growth comparable to that of the recipient parent K-12 strain without pU0928 or pU1284 infected at an MOI of 0.01. Lust infection of the pU0928 and pU1284 transconjugants gave results similar to those for P1vir at the MOI tested (Fig. 2C and Fig. S2C). In contrast, the pU1223 transconjugants had results comparable to those for the naive recipients, indicating that pU1223 does not change growth (Fig. 2A to C and Fig. S2A to C).

Except for ampicillin (Amp) and spectinomycin (Spec) resistance in UMB1091, the transconjugants exhibited growth on antibiotic plates similar to that of the respective plasmid donor urinary microbiota strain (Table S3). The stability of pU0928 was tested by incubation for multiple days in LB without the antibiotic that selects for the plasmid (i.e., tetracycline). After 10 days, the growth on LB plates was comparable to the growth on LB plates containing tetracycline (Fig. 2D). Cultures grown overnight from days 1 and 10 were used to grow the bacteria on antibiotic plates and perform a phage spot titration assay. The growth on antibiotic plates and phage permissivity profiles did not differ between day 1 and day 10 (Table 2).

**TABLE 2:** Phage spot titration and antibiotic plate growth phenotypes (overnight compared to incubation for 10 days)^*a*^

Strain	Conjugated plasmid	Phage spot titration				Antibiotic plate growth phenotype					
		No. of days of passage	P1vir spot titration	Greed spot titration	Lust spot titration	LB	Ampicillin	Chloramphenicol	Kanamycin	Spectinomycin	Tetracycline
K-12 MG1655	None	1	8	8	8	+	−	−	−	−	−
K-12 MG1655	None	10	8	8	8	+	−	−	−	−	−
UMB0928	pU0928	1	No spot	No spot	No spot	+	+	−	−	−	+
UMB0928	pU0928	10	No spot	No spot	No spot	+	+	−	−	−	+
K-12 MG1655 *yfiQ*::Cm Δ*cobB*	pU0928	1	3	3	3	+	+	+	−	−	+
K-12 MG1655 *yfiQ*::Cm Δ*cobB*	pU0928	10	3	3	3	+	+	+	−	−	+
K-12 MG1655/pCA245n-cm	pU0928	1	3	3	3	+	+	+	−	−	+
K-12 MG1655/pCA245n-cm	pU0928	10	3	3	3	+	+	+	−	−	+

a Numbers denote the titration of phage (starting at 10^9^ PFU/mL, which is titration 1) at which lysis was observed on an E. coli lawn (~10^8^ CFU/mL). A lower number indicates lysis observed only at lower titrations (i.e., higher phage PFU per milliliter). “+” denotes growth on an antibiotic medium plate. pU0928 has a native tetracycline resistance cassette. Chloramphenicol resistance was used as a marker in K-12 strains during conjugation assays.

### Analysis of urinary plasmid sequences in E. coli K-12 transconjugants.

Of the plasmidic assemblies tested, pU0928 and pU1284 reduced permissivity to phage infection; in contrast, pU1091, pU1223, and pU6721 did not change the infection phenotype relative to the naive recipients. The transconjugants were subjected to both short- and long-read sequencing; all transconjugants except the one carrying pU1091 had long-read-sequenced contigs (Table 3); the plasmid contigs had sequence similarity (>87% query coverage and >99% identity) to plasmid entries in the NCBI database. The transconjugant carrying pU0928 was predicted to possess 2 circular plasmids (contig 6 and contig 11); only contig 11 had *incF* genes (Table 3). The transconjugant carrying pU1223 was predicted to possess one circular plasmid (contig 2) with *incF* genes (Table 3). The transconjugant carrying pU1284 was predicted to possess two circular plasmid contigs, but only contig 1 had *incF* (Table 2). The transconjugant carrying pU6721 was predicted to possess 3 circular plasmids (contigs 19 to 21), with only contig 20 having *incF* genes (Table 3). For all plasmid contigs, only those with *incF* genes were predicted to carry antibiotic resistance. None of the plasmid contigs within a single transconjugant shared any replicon genes. Virulence genes were predicted to be present in both IncF and non-IncF plasmids.

**TABLE 3:** Overview of urinary plasmids conjugated into E. coli K-12

Plasmid donor	Plasmid contig sequenced	Topology	Length (bp)	Replicon(s)	Antibiotic resistance genes	Antibiotic resistance(s) predicted	Virulence gene(s)
UMB0928	6	Circular	86,594	IncI1-I(Alpha)	None		*cia*
UMB0928	11	Circular	102,837	Col156, IncFIA, IncFIB(AP001918), IncQ1	*tet*(B), *sul2*, *dfrA17*, *bla*_TEM-1B_	Tetracycline, sulfamethoxazole, trimethoprim, β-lactam, aminoglycoside	*senB*
UMB1223	2	Circular	154,298	Col156, IncFIA, IncFIB(AP001918), IncFII(pRSB107)	*aph(3″)-Ib*, *aph(6)-Id*, *aph(6)-Id*, *aph(3″)-Ib*, *mph*(A), *sul2 dfrA14*, *bla*_TEM-1B_, *tet*(B)	Aminoglycoside, macrolide, sulfamethoxazole, trimethoprim, β-lactam, tetracycline	*anr*, *senB*, *traT*
UMB1284	1	Circular	98,647	IncFIA, IncFII	*aadA5*, *aac(6′)-Ib-cr*, *aadA5*, *aac(6′)-Ib-cr*, *sul1*, *dfrA17*, *tet*(A), *bla*_CTX-M-15_, *bla*_OXA-1_, *mph*(A), *qacE*, *catB3*, *catB3*	Aminoglycoside, aminocyclitol, quinolone, macrolide, sulfamethoxazole, trimethoprim, β-lactam, tetracycline, quaternary ammonium compound, amphenicol	*traT*
UMB1284	7	Circular	35,494	IncX4	None		None
UMB6721	19	Linear	84,320	IncB/O/K/Z	None		*traT*
UMB6721	20	Linear	136,779	Col156, IncFIB(AP001918), IncFII(29)	*dfrA17*, *sul1*, *tet*(B), *qacE*, *aadA5*, *aadA5*	Trimethoprim, sulfamethoxazole, tetracycline, quaternary ammonium compound, aminocyclitol, aminoglycoside	*senB*, *traJ*, *traT*
UMB6721	21	Circular	32,089	IncX4	None		None

The transconjugant plasmid contigs were reviewed for genes that may antagonize phage infection and thus explain the change in the phage infection phenotype (Table 4). Plasmid pU0928 is predicted to carry the immunity (*imm*) gene characterized in phage T4 and involved in phage superinfection exclusion (46, 47). All transconjugants were predicted to possess the *traT* gene reported to block phage adsorption, although the phage-permissive plasmid pU1223 also had this gene (16, 19, 20). All transconjugants were predicted to carry the TA modules *pemIK* and *ccdAB*. In addition, we identified phage-associated ORFs that encoded a phage integrase and a dihydrofolate reductase enzyme. Given that these genes are associated with prophages, the plasmid contigs were scanned for other phage-like sequences (Table S4). There were putative phage-like sequences present in the majority of the plasmid contigs (Fig. S4). pU1223 contig 2 shared a phage hit with pU1284 contig 1, but otherwise, all phage hits were distinct, with various degrees of completeness.

**TABLE 4:** Coding regions in urinary plasmids with functions linked to phage infection^*a*^

Predicted coding region	Predicted gene function(s)	UniProt accession no.	K-12 less permissive to phage with plasmid		K-12 permissive to phage just like the WT	
			pU0928	pU1284	pU1223	pU6721
CcdA	Toxin of the *ccdAB* module	P62552	+	+	+	+
CcdB	Antitoxin of the *ccdAB* module	P62554	+	+	+	+
Dihydrofolate reductase	Phage DNA synthesis and protein assembly	P27422	+	+	−	+
Imm	Phage superinfection exclusion	Q03708	+	−	−	−
PemI	Antitoxin of the *pemIK* module	P13975	+	+	+	+
PemK	Toxin of the *pemIK* module	P13976	+	+	+	+
Phage integrase	Integrates phage genetic material into the host genome	P62590	+	+	+	+
TraT	Blocks phage adsorption	B1VCB1	+	+	+	+

a “+” denotes the presence of amino acid sequence query coverage and identity of >90%.

## DISCUSSION

Plasmids and phages are MGEs that impose distinct selective pressures on bacteria (11, 29, 43, 45). We understand more of the complexity of plasmid and phage dynamics but less of the role that plasmids may play in phage predation of E. coli, such as those present in the urobiota (13, 28, 45). E. coli is the urinary bacterial species most associated with UTI; its management can be difficult due to virulence factors and antibiotic resistance, often encoded by plasmids (30, 40, 48, 49). Of particular interest, F plasmids in E. coli are easily transmissible and persistent and often carry antibiotic resistance and virulence traits (19, 41, 50). In characterizing urinary E. coli plasmids, we paid special attention to profiling genes involved in plasmid retention (i.e., TAs), antibiotic resistance, and virulence (40).

During the initial course of this project, short-read whole-genome sequencing was utilized to sequence and separate genetic regions of the E. coli genome that were plasmidic, which were then analyzed as a unit to identify the best candidates for conjugation into E. coli K-12. Plasmidic assemblies with *incF* genes were predicted to have multiple antibiotic resistance genes and thus to grow on multiple types of antibiotics (Fig. 1B; see also Fig. S1A to C in the supplemental material). In a previous study, we catalogued conjugation systems in urinary E. coli isolates, and we used this information here to transfer plasmids from urinary strains (UMB0928, UMB1223, UMB1091, UMB1284, and UMB6721) to E. coli K-12 strain MG1655 derivatives (Table S3) (38). Rather than modifying the urinary plasmids, here, we used native antibiotic resistance on urinary plasmids as a selection marker during the experiments. For pU0928, its multiple antibiotic resistances were stably maintained in MG1655 derivatives for 10 days, even in the absence of selection (i.e., passaged on LB), which is potentially explained by the TA modules *pemIK* and *ccdAB* (Fig. 2D) (22, 51, 52). TA modules, the most common being *mazEF* and *pemIK*, were present in urinary plasmids (Fig. 1A). Virulence genes were also present in the IncF group in a higher proportion and diversity than in the non-IncF group (Fig. 1C). After the conjugation of urinary plasmids into an MG1655 derivative, virulence genes were then detected in the transconjugants. Taken together, these data show not only that urinary plasmids carry antibiotic resistance and virulence genes but also that these genes can be transferred via conjugation to a naive strain and stably maintained, potentially due to TA modules, a relevant factor for E. coli populations in the urinary tract (22, 53). Following this profiling of urinary plasmids, the key unanswered question was, How do these plasmids interact with phage?

We showed evidence that two E. coli K-12 transconjugants (i.e., those carrying pU0928 and pU1284) could decrease a naive host’s permissivity to phage infection (Fig, A to C, Tables 1 and 2, and Fig. S2) but could not provide immunity, as a high titer of phage (e.g., 10^10^ PFU/mL of P1vir; MOI of 10^2^ PFU/mL) could still result in lysis but not lower titers. In contrast, the urinary E. coli strains used as plasmid donors were not lysed even at the highest concentration of phage tested. This plasmid-borne protective effect was observable not just in transconjugant cultures grown overnight but also even after passaging of the transconjugants for 10 days in the absence of plasmid selection (Table 2 and Fig. 2D). These results included testing with the lytic urinary phages Greed and Lust. While not thoroughly studied in the urinary tract, phage titers in natural environments have been estimated to be 10^7^ PFU/mL in soil samples and range from 10^2^ to 10^5^ PFU/mL in marine samples (54–56). Therefore, we can infer that in low-biomass environments such as the urinary tract, these phage-nonpermissive plasmids could provide an advantage to E. coli under phage predation (8). Phage predation could be a selective force for phage-nonpermissive plasmids in urinary bacteria (13, 14, 44). This scenario is akin to antibiotic utilization in the presence of bacteria carrying resistance plasmids: bacteria with plasmid-borne antibiotic resistance will survive, propagate, and, thus, increase the frequency of the plasmid (23, 43, 57).

That stated, the mechanism by which the urinary plasmids protect urinary E. coli from phage predation remains unknown. Given our results, we know that traits expressed by the urinary plasmid did not confer immunity (i.e., zero permissivity to infection) but rather provided protection below a given MOI. Rather than plasmids granting infection immunity, akin to a phage receptor mutation, our data support a stoichiometric relationship between the plasmid’s mechanism and infecting phage particles (Fig. 2A to C, Table 1, and Fig. S2). In their study of bacterium-plasmid-phage interactions, Harrison et al. posited that conditions that limit extinction may stabilize phage-bacterium coexistence (45). In this scenario, a mechanism that reduces permissivity to infection may be more stable in the long term than one that attempts infection immunity (28). In terms of mechanism, we must highlight that phage permissivity was relatively comparable whether transconjugants were infected with P1vir, Greed, or Lust. P1vir is a temperate *Myoviridae* phage (genus *Punavirus*) modified to undergo only the lytic life cycle; its structure consists of an icosahedral head connected to a tail with six tail fibers (33, 58, 59). Greed and Lust are from the *Siphoviridae* family (genus *Seuratvirus*) and are related to the phages Seurat and CAjan but are still genetically distinct from each other; both phages were noted upon transmission electron microscopy to have a head connected with a tail, with tail fibers predicted in their genomes (32, 33, 60). We hypothesize that the mechanism that decreases infection permissivity does not target a factor exclusive to a single phage but potentially is a conserved mechanism in the three phages, perhaps related to adsorption or the lytic pathway (14, 61, 62).

To better understand the plasmids in the K-12 transconjugants, we sequenced the genetic contents of the transconjugants via long-read sequencing and analyzed the plasmid sequences. Plasmid contigs were predicted in transconjugants carrying plasmidic assemblies pU0928, pU1223, pU1284, and pU6721, with pU0928, pU1284, and pU6721 possessing multiple circular contigs. A pertinent question is whether each plasmid contig represents an individual plasmid. Each contig had high query coverage and sequence identity to plasmid sequences in the NCBI database, and all but pU1284 contig 19 were predicted to be circular. Contigs within a single transconjugant did not share replicons, indicating that if these contigs are separate plasmids, they would be stably maintained (Table 3). All transconjugants were predicted to have one contig with *incF* genes, and IncF contigs had putative antibiotic resistance genes. Multiple genes with functions that antagonize phage infection were identified in the transconjugants’ plasmid contigs, but outside *imm* in pU0928, there was no gene(s) that was present only in pU0928 and pU1284 and absent from PU1223 and pU6721 (Table 4) (19, 22, 47).

In the transconjugants, we identified genes encoding a phage integrase and the enzyme dihydrofolate reductase, genes that are linked to phage biology (63–66). Certain phages such as Lambda utilize phage integrases to integrate their genome into the host genome (34, 67). A phage integrase is a signature for prophages but by itself does not indicate viability (64, 68, 69). Dihydrofolate reductase reduces dihydrofolic acid to tetrahydrofolic acid, which is in involved in the synthesis of amino acids and nucleic acids (65). In phages, this enzyme plays a role in DNA synthesis but has also been linked to the proper packaging of the capsid; this enzyme must be finely regulated to achieve the proper coordination of the phage life cycle (65, 66). This enzyme can be crucial such that some phages encode their own dihydrofolate reductase, which replaces the host enzyme during the infection process (66). Potentially, the dihydrofolate reductase in the plasmid could compete or otherwise interfere with the propagation of invading phages (70).

**FIG 3:**
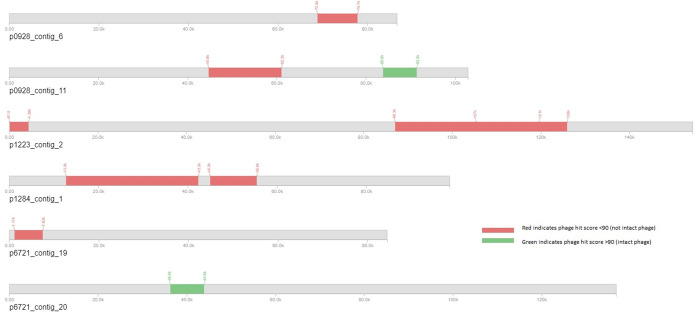
Comparison of phage sequences predicted in pU0928, pU1223, pU1284, and pU6721. Transconjugant plasmid contigs were scanned with PHASTER to identify phage sequences. Contig 11 in pU0928 and contig 20 in pU6721 are predicted to be intact phages.

Given that phage integrases are associated with prophages, we scanned the transconjugant contigs for phage sequences (Fig. 3). All transconjugants were predicted to contain phage sequences with different degrees of completeness (Table S4) (71). The lower completeness score could indicate that, rather than having a complete prophage, plasmids could have acquired phage-like genes or that these are remnants of past phage integrations (72, 73). There have been reports of prophages integrating into plasmids, and prophage superinfection immunity and exclusion have been documented, but to our knowledge, there are no reports of urinary plasmids protecting E. coli from phage infection (46, 70, 74). Future studies should focus on identifying the genes responsible for the phage-nonpermissive mechanism in F plasmids. Fortunately, this may be a realistic endeavor given that pU0928 and pU1284 are now in the genetically tractable E. coli K-12 MG1655 (71).

### Phage and urinary E. coli plasmid interactions in the urinary tract.

Extrapolating from our results and what we know of other niches, we can estimate that plasmids are widespread in urinary E. coli strains (41, 75, 76). We propose a scenario that parallels how antibiotic use can select for bacteria hosting plasmids with antibiotic resistance genes (44, 77, 78). Phage predation in the urinary tract may drive the transmission and persistence of phage-nonpermissive plasmids and, by extension, the genes carried by these plasmids, such as antibiotic resistance and virulence genes. When a urinary E. coli strain is exposed to coliphage, it can defend itself with phage-nonpermissive genes in its chromosome, plasmids, or prophage (79). Chromosomal genes may have limits on the content that can be mutated, while prophages may require lytic activation for rapid propagation in the host population (45, 61, 80). However, an advantage of plasmids is that they are pliable, nonessential MGEs that can be transmitted vertically and horizontally without fatally disrupting the host (81, 82).

A major clinical issue is the increasing frequencies of antibiotic resistance and virulence in bacteria, both of which are traits that can be associated with plasmids (40, 83, 84). Potentially, phage could drive the retention and spread of clinically relevant plasmids in the human microbiota. In this study, we present evidence that specific urinary plasmids can reduce permissivity to coliphage infection. These plasmids are conjugable and stable and confer antibiotic resistance to the host. Virulence and antibiotic resistance are commonly associated with F plasmids, and predation by phage could be a very relevant selection factor in the maintenance and transmission of these traits. Future studies should focus on identifying the genetic determinants in plasmids that affect phage infection. Further tests should determine to what extent plasmids containing phage-protective factors can impact urinary E. coli, plasmid, and phage population dynamics.

## MATERIALS AND METHODS

### Plasmidic assembly, genomic and gene homology scans, and annotation.

We used urinary E. coli isolates and previously published sequence read data (BioProject accession number PRJNA316969) (85). Urinary isolates were previously recovered from urine samples obtained by transurethral catheterization from adult females during several institutional review board (IRB)-approved studies at Loyola University Chicago (approval numbers LU203986, LU205650, LU206449, LU206469, LU207102, and LU207152) and the University of California, San Diego (approval number 170077AW). Raw sequencing reads were assembled using plasmidspades.py of SPAdes v3.12 with k values of 55,77,99,127 and the only-assembler parameter (86, 87). Plasmidic assemblies were queried against the nonredundant/nucleotide (nr/nt) database via an NCBI (Web) BLAST search to assign contigs as either plasmidic or chromosomal (88, 89). Only contigs exhibiting sequence similarity to plasmids were examined. A homology heatmap of plasmidic assemblies was generated using sourmash v4.0 with the parameters scaled=1000 and k=31 (90). For comparison, this included three urinary Klebsiella pneumoniae plasmidic assemblies, which were processed as described above.

We catalogued the plasmidic assemblies via PlasmidFinder v2.1 using the *Enterobacteriaceae* database with minimum thresholds of a 95% identity and a 60% minimum coverage (91). Given their *inc* gene profiles, we assigned plasmids as F plasmids (IncF group) or not (non-IncF group) (40, 92). To identify known acquired antibiotic resistance genes, we scanned the urinary plasmidic assemblies with ResFinder v4.1 using the “acquired antimicrobial resistance genes” option and the Escherichia coli species database (93). To identify known virulence genes, the urinary plasmidic assemblies were scanned with VirulenceFinder v2.0 using the Escherichia coli species database, with an identity threshold of 90% and a minimum sequence length of 60% (94). Urinary plasmidic assemblies were annotated using Prokka v1.14.5 and sorted by length via the sortbyname.sh script from bbmap (95, 96). Amino acid ORFs were clustered by homology using USEARCH v.11.0 with the parameters -cluster-fast -id 0.8 -clusters (97).

### Urinary plasmid conjugation and phenotype testing.

Native antibiotic resistance cassettes were identified via ResFinder as described above. To validate predictions, urinary E. coli isolates were struck onto lysogeny broth (LB) plates supplemented with the antibiotic ampicillin (Amp) (100 μg/mL), chloramphenicol (Cam) (25 μg/mL), kanamycin (Kan) (40 μg/mL), spectinomycin (Spec) (100 μg/mL), or tetracycline (Tet) (15 μg/mL). Both urinary and laboratory E. coli isolates were incubated overnight at 37°C.

To test urinary plasmid effects on phage infection permissiveness, conjugal plasmids from urinary E. coli isolates were transferred to derivatives of E. coli K-12 strain MG1655 as previously described (38, 98–100). These recipients included MG1655 transformed with multicopy plasmid pCA24n, encoding chloramphenicol resistance (99–101). Other recipients were MG1655 with deletions of the *yfiQ* and/or *cobB* gene marked by a resistance cassette. In E. coli, YfiQ is a lysine acetyltransferase and CobB is a lysine deacetylase; these gene mutations were used because they exert no phenotype under the growth conditions tested (102). Plasmid pCA24n-*yfiQ* was purified from the ASKA collection (101).

The lytic phages P1vir, Greed, and Lust were described previously (32, 33). We determined their titers using the full-plate titer technique (103) and tested the permissibility of E. coli isolates with the following phage spot titration assay. Briefly, E. coli transconjugants and controls were struck from frozen stocks onto selective plates and incubated overnight at 37°C. Single colonies were inoculated into 5 mL LB with appropriate selection and aerated overnight at 37°C. From each culture grown overnight, 100 μL was transferred to 5 mL of liquid LB with the appropriate antibiotic and aerated at 37°C until early exponential phase (optical density at 660 nm [OD_660_] of ~0.4; ~3 × 10^8^ CFU/mL). From each subculture, 200 μL was transferred to a tube with 0.7% agar LB medium preheated to 52°C, immediately mixed, and plated onto a 1.5% agar LB plate. Plates were cooled for 10 min and spotted with 10 μL of each phage as a 1:10 serial dilution in LB with a starting concentration of 10^10^ PFU/mL and a final concentration of 10^2^ PFU/mL. Phage spots were dried for 20 min, and the plates were incubated overnight at 37°C. The following day, the lowest titration that resulted in clearance was noted; an integer was given to each titration based on the number of dilutions that it was removed from the starting concentration (with the lowest titration being 1 dilution at 10^9^ PFU/mL and the highest being 8 dilutions at 10^2^ PFU/mL). A control plate consisted of an MG1655-derived lawn (200 μL of ~3 × 10^8^ CFU/mL) spotted with P1vir, Greed, and Lust (10 μL of 10^10^ PFU/mL) plus the negative controls (10 μL of LB and 10 μL of temperate phage Lambda).

To further assess the effect of urinary plasmids on the phage permissiveness of the transconjugants, growth curves were performed. Transconjugants and controls were struck from frozen stocks onto the appropriate antibiotic plates and incubated overnight at 37°C. Single colonies were inoculated into 10 mL LB with the appropriate antibiotic and aerated overnight at 37°C. From each culture grown overnight, 1 mL was transferred to 25 mL of liquid LB in a 250-mL flask to obtain approximately the same cell density (OD_660_ of ~0.2); subcultures were aerated at 37°C until early exponential phase (OD_660_ of ~0.4). Each phage (P1vir, Greed, and Lust) was titrated, and 0.5 mL was added to the flask to achieve a multiplicity of infection (MOI) of 0.01 or 10.0, with a no-phage control. Cultures were aerated at 37°C for 8 h, with the OD_660_ being measured every hour. Each treatment was repeated in biological triplicate.

To test antibiotic resistance after conjugation, the transconjugants, their urinary plasmid donor isolates, and their naive recipients were grown on antibiotic plates (tetracycline, kanamycin, ampicillin, spectinomycin, chloramphenicol, and the LB control), as described above. To test plasmid stability, urinary isolate UMB0928, the recipients MG1655/pCA24 and MG1655 *yfiQ*::Cm, and one transconjugant of MG1655/pCA24 were aerated at 37°C in 5 mL of liquid LB in the absence of antibiotic selection for plasmid pU0928 for 10 days. Cultures were plated onto tetracycline (pU0928 selection marker) and LB plates in triplicate daily and incubated at 37°C overnight, and the colonies were counted. Plasmid stability was calculated as the number of colonies on the tetracycline plate over the number of colonies on the LB plate. A ratio of 1 indicates plasmid retention, while a ratio of 0 indicates plasmid loss. To assess phage permissivity after incubation in the absence of selection, on days 1 and 10, the isolates were grown overnight to perform a spot titration assay and struck onto antibiotic plates, as described above.

### Urinary plasmid extraction, sequencing, and analysis.

We extracted and sequenced the genomes from the transconjugants, as described previously (37); sequencing was performed by both short- and long-read sequencing. Plasmid analysis of the transconjugants was performed, as described above, using the long-read contigs. The curated urinary plasmid sequences obtained from the transconjugants using the above-described extraction and sequencing procedures were scanned for phage genetic content via PHASTER using default settings (104).

### Data availability.

Sequence data for the urinary E. coli isolates can be found under BioProject accession number PRJNA316969. Long-read sequence data and plasmid assemblies for the E. coli K-12 transconjugants can be accessed under BioSample accession numbers SAMN33025487 for p0928, SAMN33025490 for p1091, SAMN33025488 for p1223, SAMN33025491 for p1284, and SAMN33025489 for p6721.
