# Supplementary figures and images for: Urinary Plasmids Reduce Permissivity to Coliphage Infection
Source: Microbiol Spectr. 2023 Jul 6;11(4):e01309-23. doi: 10.1128/spectrum.01309-23 (PMC10433841; doi:10.1128/spectrum.01309-23)

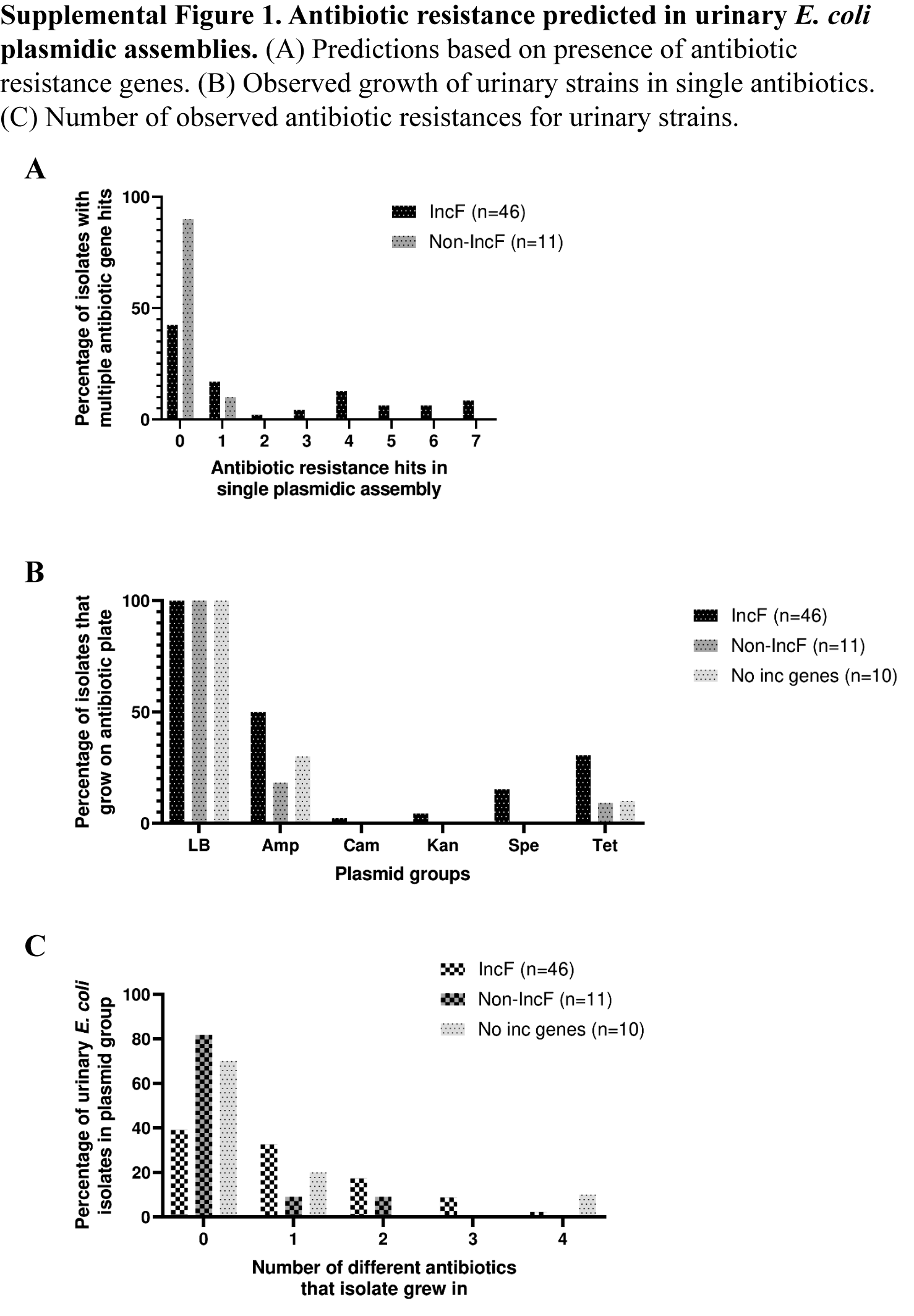

Supplement: Supplemental file 1 — Supplemental material. Download spectrum.01309-23-s0001.tif, TIF file, 0.5 MB [file spectrum.01309-23-s0001.tif]

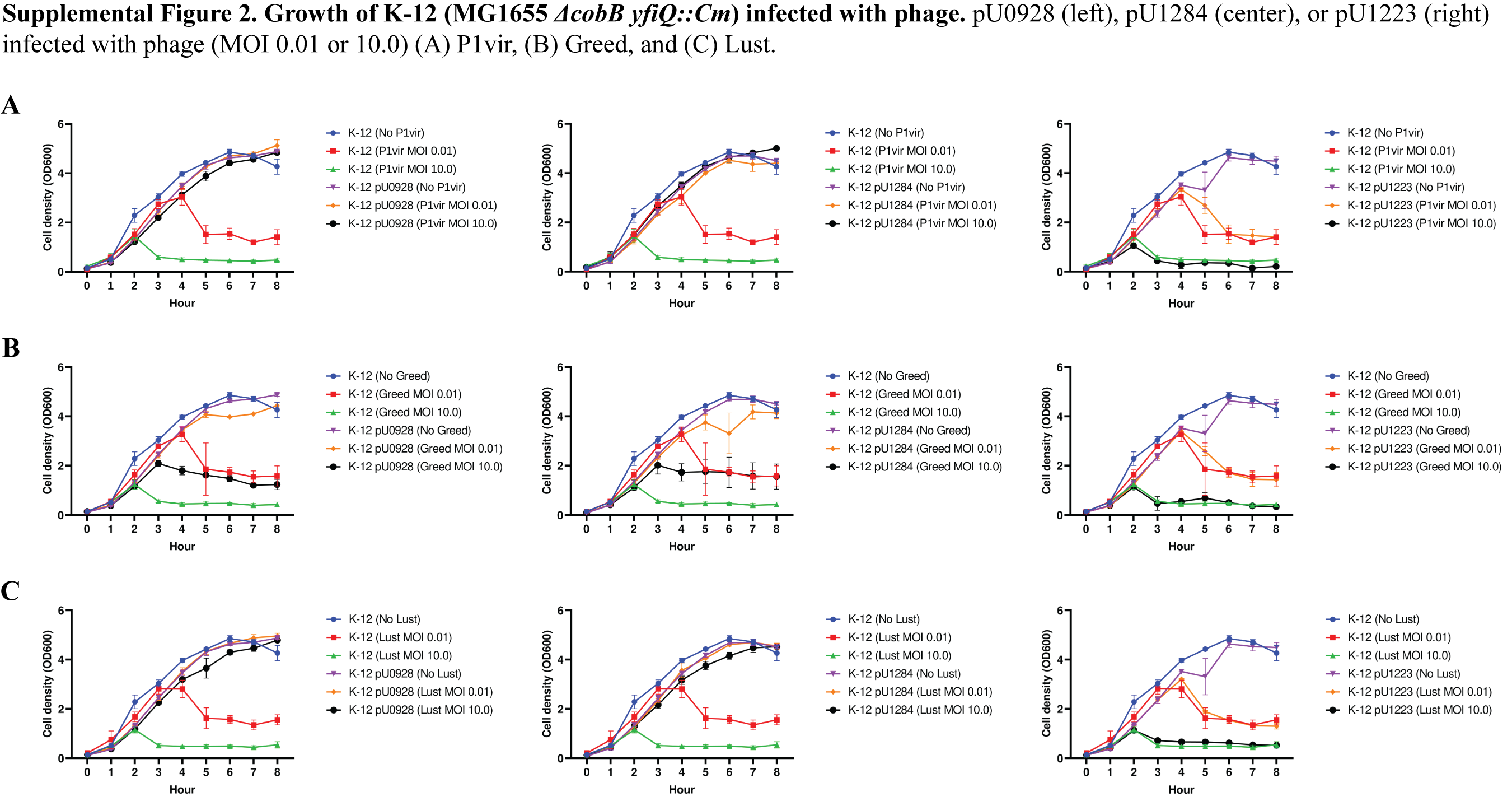

Supplement: Supplemental file 2 — Supplemental material. Download spectrum.01309-23-s0002.tif, TIF file, 2.2 MB [file spectrum.01309-23-s0002.tif]
