# Supplementary material for: Urinary Plasmids Reduce Permissivity to Coliphage Infection
Source: Microbiol Spectr. 2023 Jul 6;11(4):e01309-23. doi: 10.1128/spectrum.01309-23 (PMC10433841; doi:10.1128/spectrum.01309-23)
Supplement: Supplemental file 3 — Supplemental material. Download spectrum.01309-23-s0003.docx, DOCX file, 0.03 MB [file spectrum.01309-23-s0003.docx]

**Supplemental Table 1. Urinary *E. coli* plasmidic short-read assembly overview.**

| Strain | Contigs | Bases | ORF |
| --- | --- | --- | --- |
| UMB0103 | 12 | 231531 | 251 |
| UMB0149 | 12 | 107939 | 115 |
| UMB0276 | 0 | 0 | 0 |
| UMB0527 | 20 | 209793 | 227 |
| UMB0731 | 3 | 120907 | 149 |
| UMB0906 | 16 | 164812 | 185 |
| UMB0923 | 3 | 125070 | 142 |
| UMB0928 | 17 | 183351 | 202 |
| UMB0931 | 19 | 372241 | 463 |
| UMB0933 | 13 | 222613 | 256 |
| UMB0934 | 16 | 166402 | 202 |
| UMB0939 | 3 | 21775 | 25 |
| UMB0949 | 12 | 137266 | 156 |
| UMB1012 | 15 | 156824 | 184 |
| UMB1091 | 39 | 253344 | 300 |
| UMB1093 | 23 | 220386 | 237 |
| UMB1160 | 10 | 140088 | 172 |
| UMB1162 | 11 | 291702 | 322 |
| UMB1180 | 1 | 4990 | 5 |
| UMB1193 | 10 | 151593 | 186 |
| UMB1195 | 1 | 94010 | 107 |
| UMB1202 | 2 | 137792 | 147 |
| UMB1220 | 0 | 0 | 0 |
| UMB1221 | 3 | 139286 | 151 |
| UMB1223 | 12 | 139637 | 156 |
| UMB1225 | 0 | 0 | 0 |
| UMB1228 | 3 | 42562 | 54 |
| UMB1229 | 10 | 152983 | 189 |
| UMB1284 | 2 | 111211 | 129 |
| UMB1285 | 4 | 154985 | 159 |
| UMB1335 | 34 | 200689 | 233 |
| UMB1337 | 32 | 198104 | 233 |
| UMB1346 | 2 | 60482 | 73 |
| UMB1347 | 2 | 60482 | 73 |
| UMB1348 | 8 | 162084 | 195 |
| UMB1354 | 0 | 0 | 0 |
| UMB1356 | 0 | 0 | 0 |
| UMB1358 | 0 | 0 | 0 |
| UMB1359 | 0 | 0 | 0 |
| UMB1360 | 6 | 124614 | 195 |
| UMB1362 | 12 | 87221 | 95 |
| UMB1526 | 0 | 0 | 0 |
| UMB1727 | 13 | 239487 | 296 |
| UMB2019 | 13 | 299962 | 353 |
| UMB2055 | 3 | 127588 | 150 |
| UMB2321 | 4 | 100530 | 115 |
| UMB2328 | 2 | 96141 | 108 |
| UMB3538 | 15 | 169304 | 185 |
| UMB3641 | 12 | 93840 | 98 |
| UMB3643 | 1 | 92125 | 102 |
| UMB4656 | 28 | 151223 | 172 |
| UMB4714 | 9 | 239877 | 287 |
| UMB4716 | 9 | 239813 | 287 |
| UMB4746 | 0 | 0 | 0 |
| UMB5337 | 1 | 60192 | 72 |
| UMB5814 | 0 | 0 | 0 |
| UMB5924 | 15 | 112240 | 123 |
| UMB5978 | 11 | 101237 | 120 |
| UMB6360 | 9 | 70556 | 86 |
| UMB6454 | 4 | 112999 | 131 |
| UMB6611 | 5 | 123060 | 135 |
| UMB6653 | 34 | 261301 | 302 |
| UMB6655 | 5 | 182135 | 222 |
| UMB6713 | 13 | 211037 | 232 |
| UMB6721 | 17 | 250312 | 293 |
| UMB6890 | 4 | 181439 | 223 |
| UMB7431 | 9 | 240707 | 284 |
| Mean | 10.9474 | 156243 | 181.474 |
| Median | 9 | 156243 | 151 |
| Minimum  (non-zero) | 1 | 4990 | 5 |
| Maximum | 39 | 372241 | 463 |

Note: All manually curated plasmidic assemblies had homology

to plasmid entries in the NCBI database

(query coverage 71-100% with sequence identity 96-100%)

**Supplemental Table 2. Plasmid incompatibility genes in urinary *E. coli* plasmidic assemblies.**

| Plasmid type | Inc gene | Hits in urinary *E. coli* plasmidic assemblies (n=67) |
| --- | --- | --- |
| Col | Col(BS512) | 7 |
|  | Col156 | 26 |
|  | Col(MG828) | 1 |
|  | ColRNAI | 1 |
|  | ColpVC | 1 |
|  | Col440I | 2 |
| Total | 6 | 38 |
| IncF | IncFIA | 12 |
|  | IncFIB(AP001918) | 37 |
|  | IncFII(pRSB107) | 8 |
|  | IncFII | 10 |
|  | IncFIB(pB171) | 1 |
|  | IncFII(pCoo) | 2 |
|  | IncFIC(FII) | 6 |
|  | IncFII(29) | 18 |
|  | IncFIB(H89-PhagePlasmid) | 2 |
| Total | 9 | 96 |
| Inc-other | p0111 | 5 |
|  | IncY | 4 |
|  | IncI1-I(Gamma) | 7 |
|  | IncB/O/K/Z | 7 |
|  | IncQ1 | 2 |
|  | IncP1 | 1 |
|  | IncX4 | 4 |
|  | IncX1 | 2 |
|  | IncN | 3 |
|  | IncN3 | 2 |
|  | IncB/O/K/Z | 1 |
|  | IncI2(Delta) | 2 |
| Total | 12 | 40 |

Note: Multiple plasmidic assemblies had more than one *inc* gene hit; therefore, total hits for *inc* genes exceeds total plasmidic assemblies

**Supplemental Table 3. Antibiotic plate growth for *E. coli* K-12 transconjugants.**

|  | K-12 MG1655 ΔcobB *yfiQ*::cm | UMB0928 | K-12 MG1655 ΔcobB *yfiQ*::cm pU0928 | UMB1091 | K-12 MG1655 ΔcobB *yfiQ*::cm pU1091 | UMB1223 | K-12 MG1655 ΔcobB *yfiQ*::cm pU1223 | UMB1284 | K-12 MG1655 ΔcobB *yfiQ*::cm pU1284 | UMB6721 | K-12 MG1655 ΔcobB *yfiQ*::cm pU6721 |
| --- | --- | --- | --- | --- | --- | --- | --- | --- | --- | --- | --- |
| Strain description | Plasmid recipient | Urinary plasmid donor | Transconjugant | Urinary plasmid donor | Transconjugant | Urinary plasmid donor | Transconjugant | Urinary plasmid donor | Transconjugant | Urinary plasmid donor | Transconjugant |
| LB | + | + | + | + | + | + | + | + | + | + | + |
| Tetracycline |  | + | + | + | + | + | + | + | + | + | + |
| Kanamycin |  |  |  |  |  |  |  | + | + |  |  |
| Ampicillin |  | + | + | + |  | + | + | + | + |  |  |
| Spectinomycin |  |  |  | + |  |  |  | + | + |  |  |
| Chloramphenicol | + |  | + |  | + |  | + |  | + |  | + |

Note: Chloramphenicol resistance was used for selection of MG1655 ΔcobB yfiQ::cm during the conjugation assay. Urinary plasmids had a native tetracycline resistance cassette. Assay was repeated on K-12 strain MG1655 pCA24n-cm

**Supplemental Table 4.** **Phage-like sequences predicted in contigs from K-12 urinary plasmid transconjugants.**

| Plasmid donor | Long-read contig | Region Length | Score | # Total Proteins | Region Position | Phage hit |
| --- | --- | --- | --- | --- | --- | --- |
| UMB0928 | contig6 | 9.1Kb | 40 | 16 | 70549-79696 | PHAGE_Escher_P13374_NC_018846(1) |
| UMB0928 | contig11 | 16.6Kb | 20 | 21 | 45629-62282 | PHAGE_Escher_RCS47_NC_042128(4) |
|  |  | 7.7Kb | 100 | 12 | 85551-93275 | PHAGE_Stx2_c_1717_NC_011357(4) |
| UMB1223 | contig2 | 4.3Kb | 10 | 7 | 81-4383 | PHAGE_Stx2_c_1717_NC_011357(3) |
|  |  | 32.9Kb | 40 | 14 | 88318-121252 | PHAGE_Escher_RCS47_NC_042128(3) |
|  |  | 20.8Kb | 60 | 12 | 106889-127690 | PHAGE_Escher_RCS47_NC_042128(3) |
| UMB1284 | contig1 | 30.2Kb | 40 | 28 | 12970-43235 | PHAGE_Escher_PA28_NC_041935(2) |
|  |  | 10.6Kb | 20 | 14 | 45966-56636 | PHAGE_Escher_RCS47_NC_042128(3) |
| UMB1284 | contig7 | None |  |  |  |  |
| UMB6721 | contig19 | 6.4Kb | 30 | 13 | 1171-7628 | PHAGE_Rhodoc_CosmicSans_NC_028677(1) |
| UMB6721 | contig20 | 7.7Kb | 110 | 13 | 36845-44570 | PHAGE_Stx2_c_1717_NC_011357(4) |
| UMB6721 | contig21 | None |  |  |  |  |

Note: Score of >90 is predicted to be an intact phage
